# Association between dietary magnesium intake and peripheral arterial disease: Results from NHANES 1999–2004

**DOI:** 10.1371/journal.pone.0289973

**Published:** 2023-08-11

**Authors:** Zhe Wu, Zhishen Ruan, Gang Liang, Xu Wang, Jianlin Wu, Bin Wang

**Affiliations:** 1 The First Clinical College, Shandong Chinese Medical University, Jinan, China; 2 Department of Vascular Surgery, The First Affiliated Hospital of Shandong Chinese Medical University, Jinan, China; 3 The Traditional Chinese Medicine College, Shandong Chinese Medical University, Jinan, China; University of Sydney, AUSTRALIA

## Abstract

**Background:**

Magnesium has been found to have a strong association with peripheral arterial disease (PAD), and dietary magnesium intake shows promise as a predictor of the condition. However, the relationship between low dietary magnesium intake and an increased risk of PAD remains uncertain, and further research is needed to clarify this relationship.

**Methods:**

This study analyzed data from 5969 participants in the National Health and Nutrition Examination Survey (NHANES) of the United States from 1999 to 2004. Multifactorial logistic regression models were used to investigate the association between dietary magnesium intake and PAD.

**Results:**

Participants with PAD had lower dietary magnesium intake compared to those without PAD. After adjusting for all covariates, dietary magnesium intake remained negatively associated with PAD (OR: 0.999, 95% CI: 0.998, 1.000). When dietary magnesium intake was divided into quartiles, compared with the fourth quantile, the first quartile is associated with a higher incidence rate of PAD (OR: 1.560, 95%CI: 1.019, 2.386). Subgroup analysis revealed that the link between dietary magnesium intake and PAD remained significant among specific subgroups, including non-white, never or former smokers, and non-hypertensive, non-diabetic individuals (P < 0.05).

**Conclusion:**

Our study provides evidence that dietary magnesium intake is negatively associated with the incidence of PAD. Therefore, people with inadequate dietary magnesium intake, particularly those with excessively low intake, should increase their magnesium intake to reduce the risk of PAD.

## Introduction

Peripheral arterial disease (PAD) is a chronic atherosclerotic disease that occurs primarily in the lower extremities [1]. It is characterized by pain or claudication in the legs caused by insufficient blood supply due to narrowing or even occlusion of blood-supplying arteries in the lower extremities [2]. The incidence of PAD is increasing every year as the population ages, affecting more than 200 million middle-aged and elderly people worldwide [3], and is associated with an increased risk of various adverse clinical outcomes (coronary heart disease, stroke, amputation) [3, 4]. PAD is an underdiagnosed and under-treated disease, despite its high risk [5, 6]. Its main risk factors are smoking, diabetes, dyslipidemia, and hypertension, while the exact etiology is not fully understood [7]. Preventing the occurrence of PAD is of utmost importance, given its increasing incidence and associated risks.

There has been growing attention to the potential role of magnesium in preventing and managing PAD. Magnesium is an essential trace element in the body and an important physiological regulator of vascular tone [8], which can prevent atherosclerosis by regulating endothelial cell function, reducing inflammation, and inhibiting platelet function [9]. Early studies have shown that low serum magnesium is associated with a metabolic imbalance in patients with diabetes-related PAD [10], and low serum magnesium can increase the risk of PAD [11]. However, assessing magnesium levels can be challenging, as most of it is present in cells or bone, with serum magnesium accounting for only a small fraction [12]. Magnesium is primarily consumed through the diet and magnesium deficiency is strongly associated with inadequate magnesium intake. Therefore, dietary magnesium intake is a promising predictor of PAD and has been negatively associated with various diseases in previous studies. We investigated the potential relationship between them based on the data of National Health and Nutrition Examination Survey (NHANES) from 1999–2004.

## Materials and methods

### Data source

NHANES is a nationally representative survey conducted by the National Center for Health Statistics (NCHS) in the United States [13]. The survey assesses the health and nutritional status of Americans through a series of physical examinations, laboratory tests, and interviews. The original NHANES study data were collected with ethical approval from the Institutional review board of the NCHS with informed consent from all participants. In accord with the ethics approvals, deidentified information from the NHANES program is available and free for the public. In this study, publicly available data were obtained from the NHANES records and analysed by researchers not associated with the original NHANES program. Ethics approval for this secondary analysis of publicly available data is not required. Data from the NHANES cycle conducted between 1999 and 2004 were employed in this study.

### Ankle-brachial index

The systolic blood pressure was evaluated on the brachial artery of either the right or left arm (if the measurement on the right arm may be affected). Additionally, the posterior tibial artery was used to measure the systolic blood pressure on both ankles. The systolic ankle pressure was divided by the systolic arm pressure for each side to obtain measurements for the left and right ankle brachial index (ABI). The presence of PAD was determined when the ABI was less than 0.9 in at least one leg [14].

### Dietary trace minerals

The 24-hour dietary recall method was used to estimate subjects’ dietary intake of trace minerals [15]. This subject-driven method involves collecting a short and precise list of foods and beverages that the individual digested over 24 hours. The intake of trace minerals is determined using the National Nutrition Standards Reference Database, which is maintained by the United States Department of Agriculture. Dietary data collection and nutritional content analysis are performed by trained technical personnel to ensure the authenticity and accuracy of the information. Detailed descriptions are available from the NHANES Dietary Interviewer Procedures Manual.

### Covariates

Covariates included age, gender, race (white, black, Mexican American, and other race), annual family income(≥ $20000, < $20000), education(above high school, high school or less than high school), smoking status (now, former, never), triglycerides, cholesterol, body mass index (BMI), platelets, and the presence of hypertension and diabetes. Triglycerides and cholesterol were determined by enzymatic assay, and platelet count was determined on The Beckman Coulter MAXM instrument. Hypertension was defined as a mean systolic blood pressure ≥ 140 mmHg and a mean diastolic blood pressure ≥ 90 mmHg, or physician diagnosis or use of antihypertensive medication. Diabetes was defined as fasting glucose ≥ 7 mmol/L or random glucose ≥ 11.1 mmol/L or two-hour OGTT glucose ≥ 11.1 mmol/L or glycated hemoglobin > 6.5%, or physician diagnosis or use of glucose-lowering medications.

### Statistical analysis

Extraction, merging, analysis, and graph production of the 1999–2004 NHANES data were performed by R studio (version 4.2.1), and all statistical analyses were weighted. Continuous variables that follow a Normal distribution are presented as mean ± standard error, while continuous variables that do not follow a Normal distribution are presented as median (interquartile range). Categorical variables were expressed as numbers (percentages).

## Results

### Demographic characteristics

**Fig 1:**
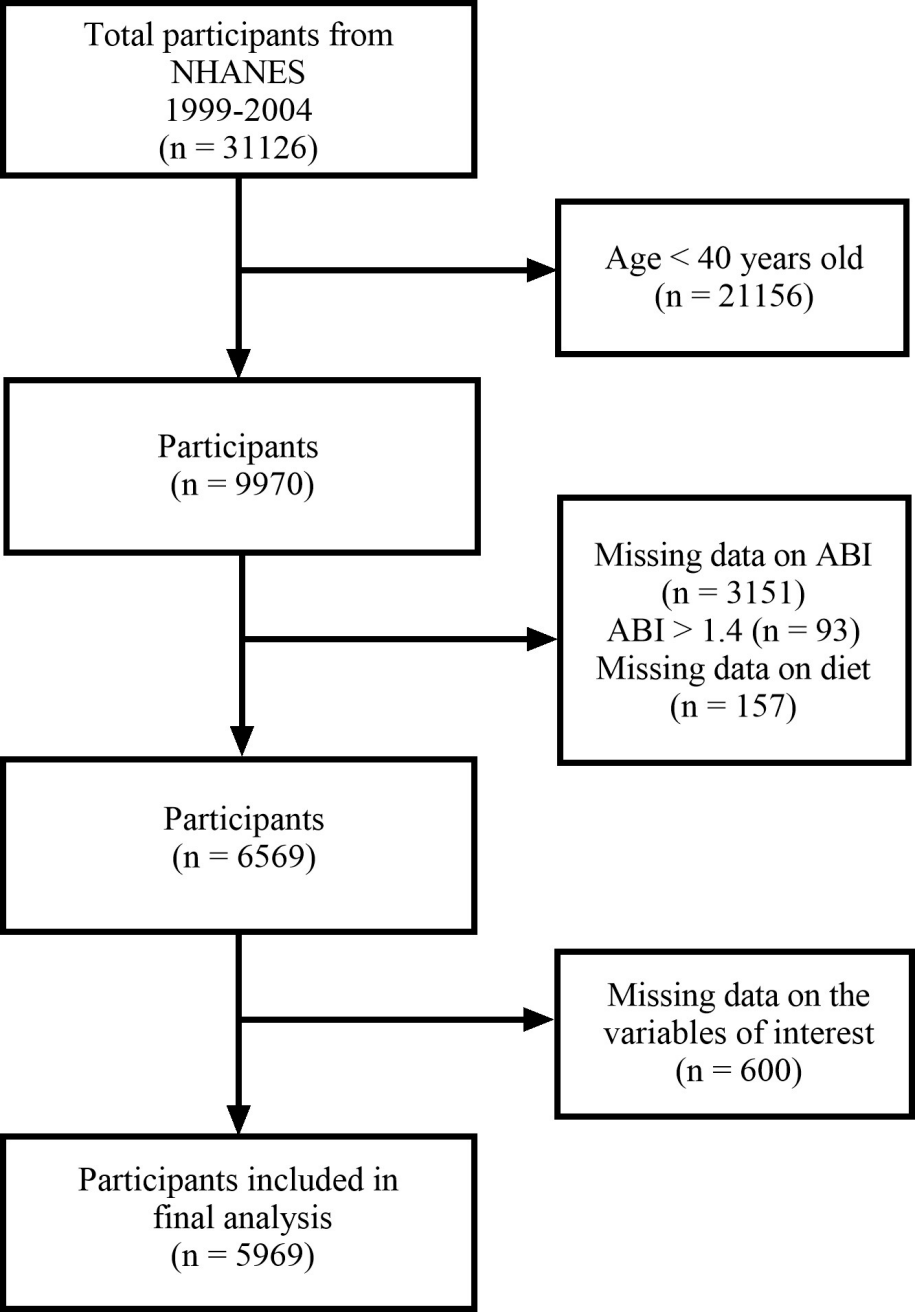
Research flowchart.

A total of 31126 participants participated in NHANES 1999–2004, participants aged 40 and above were chosen to undergo an ABI test (n = 9970). Exclusions were made for participants with missing bilateral ABI data (n = 3151), ABI values surpassing 1.4 (n = 93), or lacking diet data (n = 157). Additionally, participants with missing covariates were excluded (n = 600), resulting in a final analysis sample of 5969 participants (Fig 1).

Table 1 provides a detailed overview of the characteristics of the 5969 eligible participants who were classified into two groups based on the presence or absence of PAD. Of the total participants, 409 were in the PAD group, while 5560 were in the non-PAD group. The median age of participants in the PAD group was 68 years, significantly higher than the median age of 53 years in the non-PAD group. Moreover, the study found that the intake of both trace minerals was significantly lower in the PAD group compared to the non-PAD group (P < 0.05). This finding suggests that there may be a relationship between the intake of these trace minerals and the development of PAD. Additionally, there were significant differences between the two groups in terms of age, race, annual family income, education, cholesterol levels, smoking behavior, the presence of diabetes, and hypertension (P < 0.05).

**Table 1:** Population characteristics stratified by peripheral arterial disease.

Variable	Total (n = 5969)	Non-PAD (n = 5560)	PAD (n = 409)	*P*-value
**Age (years)**	54.00 (46.00, 64.00)	53.00 (46.00, 64.00)	68.00 (58.00, 75.00)	< 0.001
**Sex**				0.49
Female	2895 (48.50%)	2699 (50.61%)	196 (53.04%)	
Male	3075 (51.50%)	2861 (49.39%)	213 (46.96%)	
**Race**				< 0.001
White	3326 (55.72%)	3093 (78.96%)	233 (78.39%)	
Black	992 (16.62%)	901 (8.27%)	91 (14.13%)	
Mexican American	1243 (20.82%)	1176 (4.50%)	67 (3.59%)	
Other Race	408 (6.84%)	390 (8.28%)	18 (3.90%)	
**Annual family income**				< 0.001
≥ $20000	4153 (69.58%)	3943 (79.62%)	210 (61.25%)	
< $20000	1816 (30.42%)	1617 (20.38%)	199 (38.75%)	
**Education**				< 0.001
Above high school	2662 (44.6%)	2531 (56.23%)	131 (40.29%)	
High school or less than high school	3307 (55.4%)	3029 (43.77%)	278 (59.71%)	
**Dietary intake of trace elements**				
Magnesium (mg)	286.29 ± 3.50	288.30 ± 3.66	244.89 ± 6.31	< 0.001
Calcium (mg)	824.04 ± 13.69	830.42 ± 14.04	692.28 ± 30.79	< 0.001
Phosphorus (mg)	1286.18 ± 14.11	1295.85 ± 14.30	1086.88 ± 36.70	< 0.001
Iron (mg)	15.30 ± 0.19	15.38 ± 0.19	13.55 ± 0.61	0.005
Zinc (mg)	11.64 ± 0.15	11.72 ± 0.15	10.08 ± 0.54	0.004
Copper (mg)	1.29 ± 0.02	1.30 ± 0.02	1.11 ± 0.07	0.01
Sodium (mg)	3315.73 ± 34.27	3340.93 ± 34.83	2796.13 ± 129.30	< 0.001
Potassium (mg)	2817.98 ± 32.81	2832.90 ± 34.05	2510.12 ± 71.67	< 0.001
Selenium (mg)	106.28 ± 1.08	107.02 ± 1.15	90.91 ± 3.02	< 0.001
**BMI kg/m** ^**2**^	28.16 ± 0.13	28.14 ± 0.13	28.65 ± 0.39	0.21
**Triglycerides (mg/dL)**	153.30 ± 2.91	152.34 ± 3.01	173.12 ± 10.45	0.06
**Cholesterol (mmol/L)**	5.41 ± 0.02	5.42 ± 0.02	5.30 ± 0.05	0.04
**Platelets (1,000 cells/uL)**	265.43 ± 1.45	265.57 ± 1.48	262.60 ± 3.94	0.46
**Smoke**				< 0.001
Former	2033 (34.06%)	1855 (33.21%)	178 (43.46%)	
Never	2744 (45.97%)	2621 (46.27%)	123 (27.66%)	
Now	1192 (19.97%)	1084 (20.52%)	108 (28.88%)	
**Diabetes**				< 0.001
Yes	972 (16.28%)	853 (11.66%)	119 (26.43%)	
No	4997 (83.72%)	4707 (88.34%)	290 (73.57%)	
**Hypertension**				< 0.001
YES	3155 (52.86%)	2843 (45.67%)	312 (74.26%)	
No	2814 (47.14%)	2717 (54.33%)	97 (25.74%)	

### Relationship between dietary trace minerals and PAD

Using multivariate logistic regression to examine the association of trace minerals with peripheral arterial disease (Table 2), dietary magnesium, calcium, phosphorus, iron, zinc, sodium, potassium, and selenium were negatively associated with the prevalence of PAD in the unadjusted model. After fully adjusting for covariates, dietary magnesium intake remained significantly associated with the prevalence of peripheral arterial disease (OR: 0.999, 95% CI: 0.998, 1.000), whereas dietary calcium, phosphorus, iron, zinc, sodium, potassium, and selenium were not significantly associated (*P* > 0.05).

**Table 2:** Relationship between dietary trace minerals and peripheral arterial disease.

Result	Model 1		Model 2		Model 3	
	OR(95%CI)	*P*-value	OR(95%CI)	*P*-value	OR(95%CI)	*P*-value
Magnesium	0.997(0.996,0.998)	< 0.001	0.998(0.997,0.999)	0.010	0.999(0.998,1.000)	0.048
Calcium	0.999(0.999,1.000)	0.002	1.000(0.999,1.000)	0.058	1.000(0.999,1.000)	0.094
Phosphorus	0.999(0.999,1.000)	< 0.001	1.000(0.999,1.000)	0.054	1.000(0.999,1.000)	0.086
Iron	0.972(0.949,0.995)	0.019	0.982(0.960,1.005)	0.122	0.988(0.967,1.009)	0.244
Zinc	0.963(0.931,0.995)	0.027	0.986(0.954,1.018)	0.375	0.989(0.960,1.018)	0.439
Copper	0.653(0.423,1.007)	0.054	0.842(0.558,1.271)	0.404	0.922(0.641,1.326)	0.649
Sodium	1.000(1.000,1.000)	0.002	1.000(1.000,1.000)	0.147	1.000(1.000,1.000)	0.134
Potassium	1.000(1.000,1.000)	< 0.001	1.000(1.000,1.000)	0.097	1.000(1.000,1.000)	0.208
Selenium	0.995(0.991,0.998)	< 0.001	0.998(0.995,1.001)	0.119	0.998(0.995,1.001)	0.150

Adjusted variables:

Model 1: unadjusted. Model 2: age, sex, race, annual family income, education. Model 3: age, sex, race, annual family income, education, BMI, platelets, triglycerides, cholesterol, smoke, hypertension, diabetes.

OR, odds ratio; CI, confidence interval; BMI, body mass index.

### Relationship between different quartiles of dietary magnesium intake and PAD

We divide the dietary magnesium intake level into quartiles (Q1 ≤ 179.00mg, 179.00mg < Q2 ≤ 249.12mg, 249.12mg < Q2 ≤ 343.00mg, Q4 > 343.00mg). Table 3 shows that compared with the fourth quantile (Q4), the first quartile (Q1) is associated with a higher incidence rate of PAD (OR: 1.560, 95%CI: 1.019, 2.386), indicating that low magnesium intake was closely related to PAD.

**Table 3:** Relationship between different quartiles of dietary magnesium intake and peripheral arterial disease.

Variable		Model 1 OR (95% CI)	*P*-value	Model 2 OR (95% CI)	*P*-value	Model 3 OR (95% CI)	*P*-value
Magnesium	Q1 VS Q4	2.594(1.759,3.824)	< 0.001	1.806(1.164,2.800)	0.010	1.560(1.019,2.386)	0.041
	Q2 VS Q4	1.803(1.132,2.872)	0.014	1.377(0.815,2.328)	0.224	1.182(0.690,2.026)	0.530
	Q3 VS Q4	1.600(0.986,2.597)	0.057	1.318(0.810,2.144)	0.257	1.181(0.726,1.921)	0.487

Adjusted variables:

Model 1: unadjusted. Model 2: age, sex, race, annual family income, education. Model 3: age, sex, race, annual family income, education, BMI, platelets, triglycerides, cholesterol, smoke, hypertension, diabetes.

OR, odds ratio; CI, confidence interval; BMI, body mass index.

### Subgroup analysis and interaction test of dietary magnesium intake and PAD

The study conducted subgroup analysis based on age (> 60 years), gender, race, annual family income, education, current smoking status, and the presence of diabetes and hypertension. All analyses were adjusted for all covariates except for the stratified variables (Table 4). The results showed that the association between dietary magnesium intake and PAD remained significant in non-white, never or former smokers, and non-hypertensive, non-diabetic individuals (*P* < 0.05). Furthermore, interaction analysis revealed that the relationship between magnesium intake and PAD was more significant among individuals without hypertension (OR: 0.997, 95% CI: 0.995, 0.999). Additionally, there was also a potential interaction between magnesium and age.

**Table 4:** Subgroup analysis and interaction test of dietary magnesium intake and peripheral arterial disease.

Variable	OR(95% CI)	*P*-value	*P* for interaction
**Age**			0.014
> 60 years old	0.999(0.998,1.000)	0.014	
40–60 years old	0.997(0.995,0.999)	0.007	
**Sex**			0.698
Male	0.999(0.998,1.000)	0.064	
Female	0.999(0.997,1.000)	0.158	
**Race**			0.257
White	0.999(0.998,1.000)	0.129	
Non-White	0.998(0.996,0.999)	0.005	
**Annual family income**			0.811
≥ $20000	0.998(0.997,1.000)	0.017	
< $20000	0.999(0.997,1.000)	0.043	
**Education**			0.775
Above high school	0.998(0.996,1.000)	0.019	
High school or less than high school	0.999(0.997,1.000)	0.025	
**Smoke**			0.092
Now	1.000(0.998,1.001)	0.694	
Never or former	0.998(0.997,1.000)	0.005	
**Hypertension**			0.011
No	0.997(0.995,0.999)	0.001	
YES	0.999(0.998,1.000)	0.181	
**Diabetes**			0.959
No	0.999(0.997,1.000)	0.013	
YES	0.998(0.997,1.000)	0.064	

OR, odds ratio; CI, confidence interval; BMI, body mass index.

## Discussion

Using data from NHANES between 1999 to 2004, this cross-sectional study investigated the potential correlation between dietary magnesium intake and PAD. To reduce clinical confounder bias, we performed multifactorial logistic regression. At the same time, we conducted subgroup analysis and interaction tests according to age, gender, race, annual family income, education, smoking state, and the presence of diabetes and hypertension. This study found a significant inverse correlation between dietary magnesium intake and PAD risk.

Our results are in contrast to a previous study that showed no significant association between magnesium intake and PAD by analyzing the vascular screening subcohort of the Hoorn study [16]. However, the analysis based on the vascular screening subcohort had a greater selection bias because its baseline data had mostly poor vascular conditions, and the sample size of the study was small. In contrast, this study was based on the general population and used nationally representative data on middle-aged and older Americans as the sample, and the sample size was larger. Numerous studies have been conducted to explore the link between magnesium and PAD, but most of them have mainly focused on the association between serum magnesium levels and PAD. Sun et al. [11] based the Atherosclerosis Risk in Communities cohort study, which followed participants without peripheral arterial disease for more than two decades, found that participants with low serum magnesium increased the risk of developing PAD. A study by Steven Menez et al. [17] made similar results to Sun and demonstrated a consistent negative association of low serum magnesium with PAD and critical limb ischemia. Rusu’s [10] study of 114 individuals with type 2 diabetes found that their serum magnesium levels were lower, and this difference was more significant when the patients also had comorbid PAD. This is the first study to explore the link between magnesium intake and PAD in the general population.

The vast majority of magnesium intake comes from green vegetables, followed by legumes, unprocessed grains, seeds, and nuts [18]. The recommended dietary allowance (RDA) for magnesium in people over 30 years of age is 420 mg/day for men and 320 mg/day for women [19]. However, according to the data extracted from this study, the average daily magnesium intake of middle-aged and elderly people over 40 years old in the United States was only 286.29 mg/d, the average daily magnesium intake of subjects without PAD was 288.30 mg/d, and the average daily magnesium intake of patients with PAD was 244.89 mg/d, all of which were lower than the recommended intake, which shows that insufficient magnesium intake in middle-aged and elderly people in the United States is common.

Magnesium deficiency can increase the risk of PAD, which may be related to multiple factors. Magnesium is a natural calcium antagonist and has an important role in vasodilatation and increasing peripheral arterial blood flow [20]. Magnesium has a significant role in anti-atherosclerosis [21]. Magnesium can promote endothelial cell production of prostacyclin (PGI2) and nitric oxide (NO), and reduce endothelin-1 levels thus reducing vasoconstriction and promoting blood circulation [22–24]. Also, magnesium can inhibit the inflammatory response [25]. Some studies found that magnesium can reduce the concentration of inflammatory factors, such as VCAM-1, TNF-α, IL-6, and CRP [26–28]. In addition, magnesium can affect the calcium-regulated signaling pathway in the vascular wall [29], thus reducing calcium deposition and sclerosis in the vascular wall and reducing vascular calcification [30]. Also, magnesium deficiency is closely associated with dyslipidemia, metabolic syndrome, hypertension, and other high-risk factors for PAD [31].

This study also has some limitations. Firstly, magnesium intake was estimated based on the 24-hour dietary recall, which may not accurately describe long-term magnesium intake [32]. There may be differences in the magnesium content of the same food. Secondly, this study is a cross-sectional study and it is not possible to establish a definitive causal link between dietary magnesium intake and the incidence of PAD. Furthermore, the measurement of ABI was limited to participants aged 40 and above. However, among participants in the NHANES 1999–2004, participants aged 40 and above only account for about one-third of the total participants. Therefore, there may be some selection bias. Some patients with PAD under the age of 40 may be excluded, although PAD mostly occurs in older populations.

## Conclusion

There is a scarcity of research on the relationship between dietary magnesium intake and PAD. This is the first study to explore the link between magnesium intake and PAD in the general population. Our analysis revealed a significant inverse correlation between dietary magnesium intake and the prevalence of PAD. However, given the limitations of our cross-sectional study design, it is essential to conduct further prospective studies to validate these results.
